# Macrophage Plasticity in Experimental Atherosclerosis

**DOI:** 10.1371/journal.pone.0008852

**Published:** 2010-01-25

**Authors:** Jamila Khallou-Laschet, Aditi Varthaman, Giulia Fornasa, Caroline Compain, Anh-Thu Gaston, Marc Clement, Michaël Dussiot, Olivier Levillain, Stéphanie Graff-Dubois, Antonino Nicoletti, Giuseppina Caligiuri

**Affiliations:** 1 UMRS698 INSERM, Paris, France; 2 University Denis Diderot, Paris, France; 3 UMR5123 University Claude Bernard Lyon 1, Villeurbanne, France; 4 Université Pierre et Marie Curie-Paris 6, Paris, France; University of Toronto, Canada

## Abstract

As in human disease, macrophages (MØ) are central players in the development and progression of experimental atherosclerosis. In this study we have evaluated the phenotype of MØ associated with progression of atherosclerosis in the apolipoprotein E (ApoE) knockout (KO) mouse model.

We found that bone marrow-derived MØ submitted to M1 and M2 polarization specifically expressed arginase (Arg) II and Arg I, respectively. This distinct arginase expression was used to evaluate the frequency and distribution of M1 and M2 MØ in cross-sections of atherosclerotic plaques of ApoE KO mice. Early lesions were infiltrated by Arg I^+^ (M2) MØ. This type of MØ favored the proliferation of smooth muscle cells, *in vitro*. Arg II^+^ (M1) MØ appeared and prevailed in lesions of aged ApoE KO mice and lesion progression was correlated with the dominance of M1 over the M2 MØ phenotype. In order to address whether the M2->M1 switch could be due to a phenotypic switch of the infiltrated cells, we performed *in vitro* repolarization experiments. We found that fully polarized MØ retained their plasticity since they could revert their phenotype. The analysis of the distribution of Arg I- and Arg II-expressing MØ also argued against a recent recruitment of M1 MØ in the lesion. The combined data therefore suggest that the M2->M1 switch observed *in vivo* is due to a conversion of cells already present in the lesion. Our study suggests that interventional tools able to revert the MØ infiltrate towards the M2 phenotype may exert an atheroprotective action.

## Introduction

Apolipoprotein E (ApoE) knockout (KO) mice spontaneously develop atherosclerotic lesions and are widely used to study atherosclerosis. Lack of ApoE results in severe hypercholesterolemia which is necessary for the development of the disease. However, in the absence of macrophages (MØ), the severe hypercholestoremia is not sufficient to drive the pathologic process in this model [1], demonstrating that these cells exert essential functions during atherogenesis. Infiltrating MØ scavenge oxidatively-modified self-compounds accumulated in the arterial wall and are converted into foam cells, the first cells forming atherosclerotic lesions. In the last few years, it has become widely accepted that classically activated MØ (or M1) and alternatively activated MØ (or M2) are two extremes (inflammatory and reparative, respectively) of a spectrum of possible MØ phenotypes [2], [3]. A major effector molecule of inflammatory MØ is NO, which is synthesized by inducible NO synthase (iNOS) from L-arginine (L-Arg). In addition, MØ express arginases, which also catalyze L-Arg. There are two isoforms of arginase, arginase I (Arg I) is cytosolic and arginase II (Arg II) is mitochondrial. While this intracellular compartmentalization might endow them with distinct biological activities, they both transform L-Arg into L-ornithine, a precursor of polyamines and proline. Polyamines are involved in cell growth, differentiation and division whereas proline is a key component of collagen. Since a predominant expression of arginase over that of iNOS was reported in alternatively activated MØ [4] and a change in the L-Arg metabolism has been proposed to be an important component of wound healing [5], M2 MØ have been classified as reparative cells.

In the present study, we found that bone marrow-derived MØ submitted to M2 polarization conditions exclusively express the Arg I isoform and no iNOS. Conversely, M1 MØ expressed Arg II in addition to iNOS but did not express Arg I. This differential arginase isoform expression was exploited to establish the phenotype of MØ infiltrated in atherosclerotic plaques of young and aged ApoE KO mice.

Lesion-infiltrated MØ of young ApoE KO mice were virtually all Arg I+ suggesting that they were of the M2 phenotype. This type of MØ favored the proliferation of smooth muscle cells, *in vitro*. Arg II^+^ (M1) MØ appeared and prevailed in lesions of aged ApoE KO mice. Additional data suggest that this M2->M1 switch is due to a change in the cytokine milieu that triggers the conversion of the MØ already present in the lesion. Finally, lesion progression was correlated with the dominance of M1 over the M2 infiltrated MØ. These observations indicate that interventional tools able to favor the M2 MØ phenotype may exert an atheroprotective action.

## Results

### Arg I and Arg II Are Respectively Expressed by M2 and M1 Macrophages

**Figure 1:**
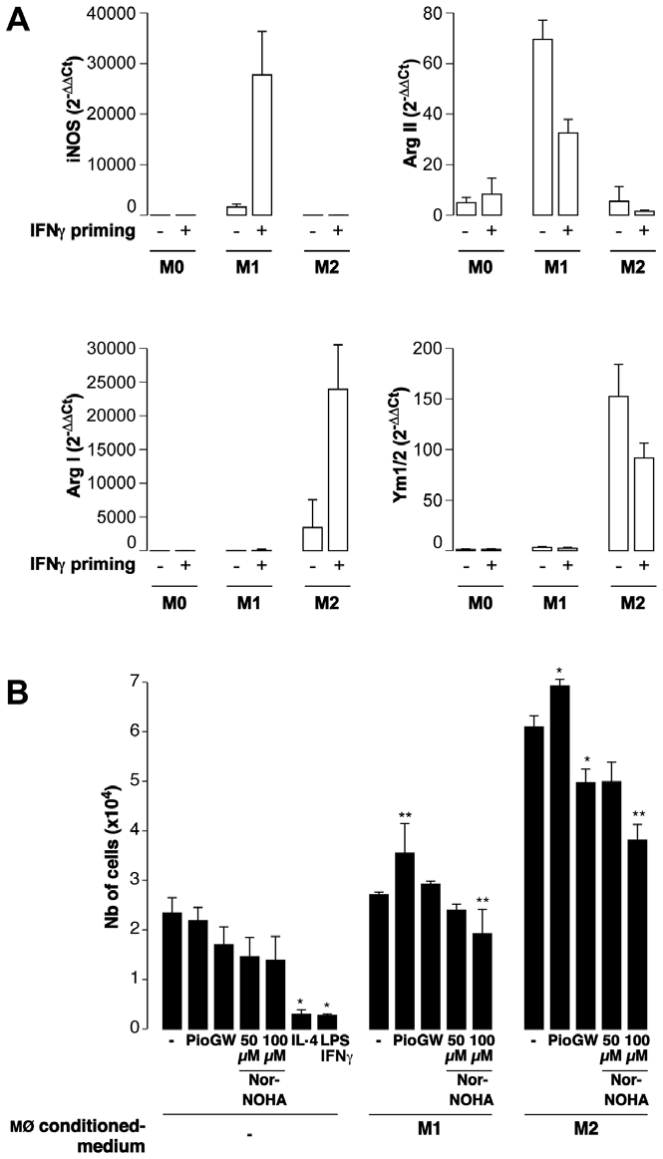
Phenotypic and functional features of M1 and M2 macrophages. **A**: The effect of an overnight IFNγ priming step was tested on C57Bl/6 mouse bone marrow-derived MØ subjected to M1- or M2-polarizing conditions. The expression of iNOS, ArgI, ArgII, and Ym1/2 were determined by real time RT-PCR on RNA extracted 10 hours after the induction of polarization. Data were calculated using the 2^−ΔΔCt^ Pfaffl formula [30] in which experimental conditions (M1 and M2) are compared to Ct values obtained in M0 MØ and normalized to the Ct values of the HPRT house-keeping gene. **B**: Primary aortic vascular smooth muscle cells (VSMCs) from C57Bl/6 mice were cultured for 48 hours in the presence of media conditioned by C57Bl/6 M1 or M2 MØ which were polarized in the presence of the PPARγ agonist pioglitazone (Pio), the PPARγ antagonist GW9662 (GW), or the arginase inhibitor Nor-NOHA. As controls, VSMCs were also cultured with the same concentration of the polarizing agents, of the PPARγ agonists and antagonists, or of the arginase inhibitor. At the end of the assay, the number of viable cells in each condition was evaluated by the MTT assay and by using a standard curve established with known numbers of cells. *; **: p<0.05; p<0.01 vs matched medium conditioned by MØ polarized in the standard way (−). Note that the number of cells obtained with the M2-conditioned medium were significantly greater than with M1-conditioned medium (p<0.01 vs matched condition).

In preliminary methodological studies, we found that bone marrow-derived MØ from C57BL/6 mice produced Arg I and Ym1/2 (a member of the chitinase family expressed by M2 MØ [6]) when subjected overnight to IL-4 (pro-M2 polarizing conditions), and Arg II after LPS stimulation (pro-M1 polarizing conditions). In these experiments, polarizing stimuli were applied on IFNγ-primed MØ as described by the group of Mosser [7]. Since this IFNγ priming step could conceivably bias the polarization, this parameter was tested (**Fig. 1A**). We found that the IFNγ priming enhanced Arg I expression in MØ subjected to M2 polarizing conditions without major changes in iNOS, Arg II and Ym1/2 expression. On the other hand, this step increased the iNOS expression of M1 polarized MØ and decreased their Arg II expression, as previously reported by Wang et al [8] (**Fig. 1A**). Non-polarized M0 MØ did not express any of these genes and the IFNγ priming step had no effect. The IFNγ priming step was used in the following experiments since it further discriminates between M0, M1 and M2 phenotypes *in vitro*.

### Medium Conditioned by M2 Macrophages Induces the Proliferation of Vascular Smooth Muscle Cells

To test whether the predominance of the arginase expression over that of the iNOS endowed M2 MØ with reparative capabilities, vascular smooth muscle cells (VSMCs) were cultured with MØ-conditioned medium. M2-conditioned medium induced the proliferation of VSMCs (**Fig. 1B**) and of adventitial fibroblasts (data not shown). By contrast, M1-conditioned medium did not affect cell proliferation (**Fig. 1B** and data not shown). The effect was not due to the presence of LPS/IFNγ or IL-4 in the conditioned media since, alone, these agents were not able to induce cell proliferation (**Fig. 1B**).

### ApoE KO Macrophages Are M2-Prone

The distinct expression of arginase isoforms by M1 and M2 macrophages could be used to establish the phenotype of macrophages infiltrated in the lesions of apoE KO mice. A prerequisite to use the arginase isoforms as M1 and M2 markers is that their distinct expression holds true for MØ of apoE KO mice. To address this issue, we explored kinetically (2, 4, 6, 24 and 48 h) the expression of Arg I, Arg II, iNOS and IL-6 in polarized bone marrow-derived MØ from C57BL/6 (MØ^B6^) and ApoE KO (MØ^ApoE^) mice.

**Figure 2:**
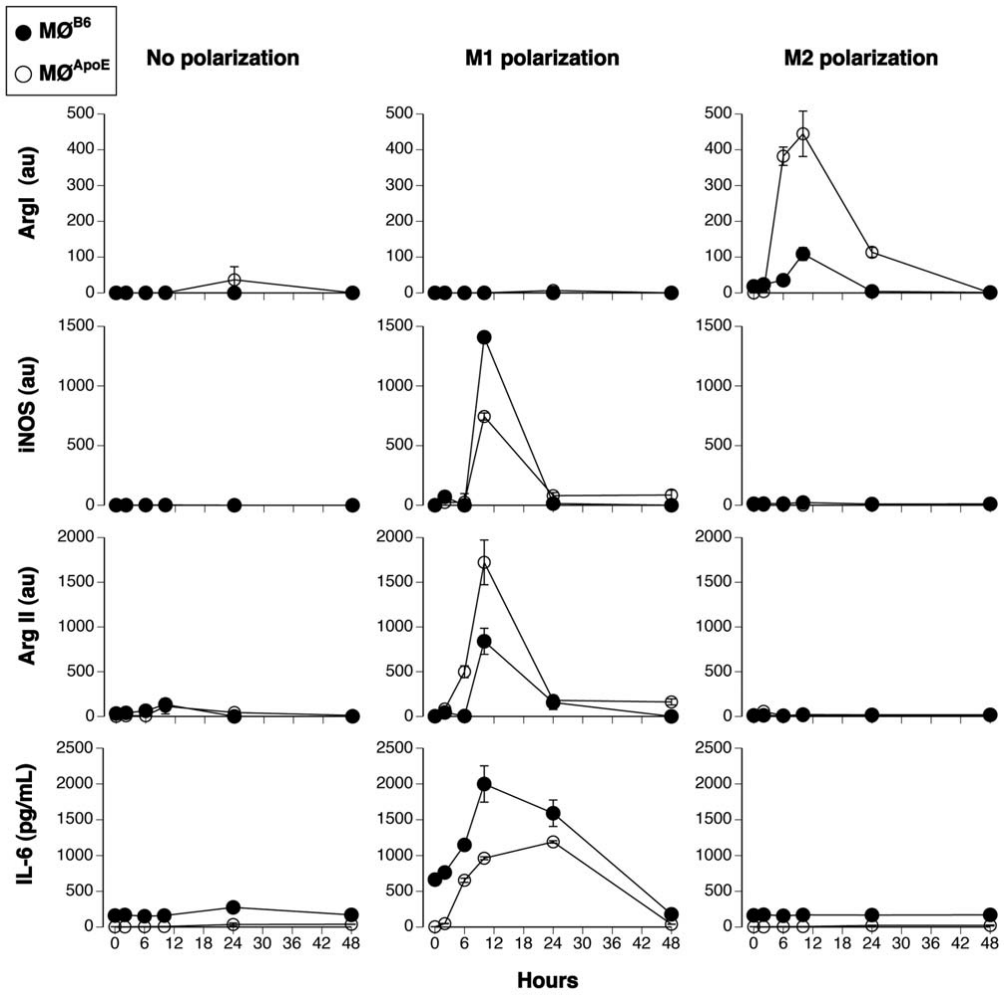
Kinetic expression of M1 and M2 markers. Bone marrow-derived MØ from C57BL/6 mice (MØ^B6^, closed circles) and ApoE KO mice (MØ^ApoE^, open circles) were differentiated, primed with IFNγ and polarized or not (No polarization) towards the M1 (M1 polarization) or the M2 phenotype (M2 polarization). Expression of Arg I, iNOS, Arg II were determined by real time PCR and normalized by HPRT (au: arbitrary unit). IL-6 was monitored in cell culture supernatant by ELISA. Results are representative of three independent experiments.

As expected, the transcription of iNOS and Arg II, but not of Arg I, and the production of IL-6 were observed in M1-polarized MØ^B6^, with a peak expression at 10 hours (**Fig. 2**). In contrast, no IL-6 was produced by M2-polarized MØ and Arg I was the only transcript observed in M2-polarized MØ^B6^, exhibiting similar kinetics (**Fig. 2**).

Lack of ApoE influenced the quantitative rather than the qualitative response to the polarizing agents. M1 MØ^ApoE^ displayed global (Arg II + iNOS) M1 gene expression levels close to those of MØ^B6^ (**Fig. 2**). The high expression of Arg II in M1 MØ^ApoE^ confirmed that it can be a reliable marker of the M1 phenotype in atherosclerotic lesions. MØ from ApoE KO mice appeared to be M2-prone since M2 polarization induced a markedly higher Arg I expression in MØ^ApoE^ compared to MØ^B6^.

### Macrophage Phenotypes in Early and Advanced Plaques in ApoE KO Mice

**Figure 3:**
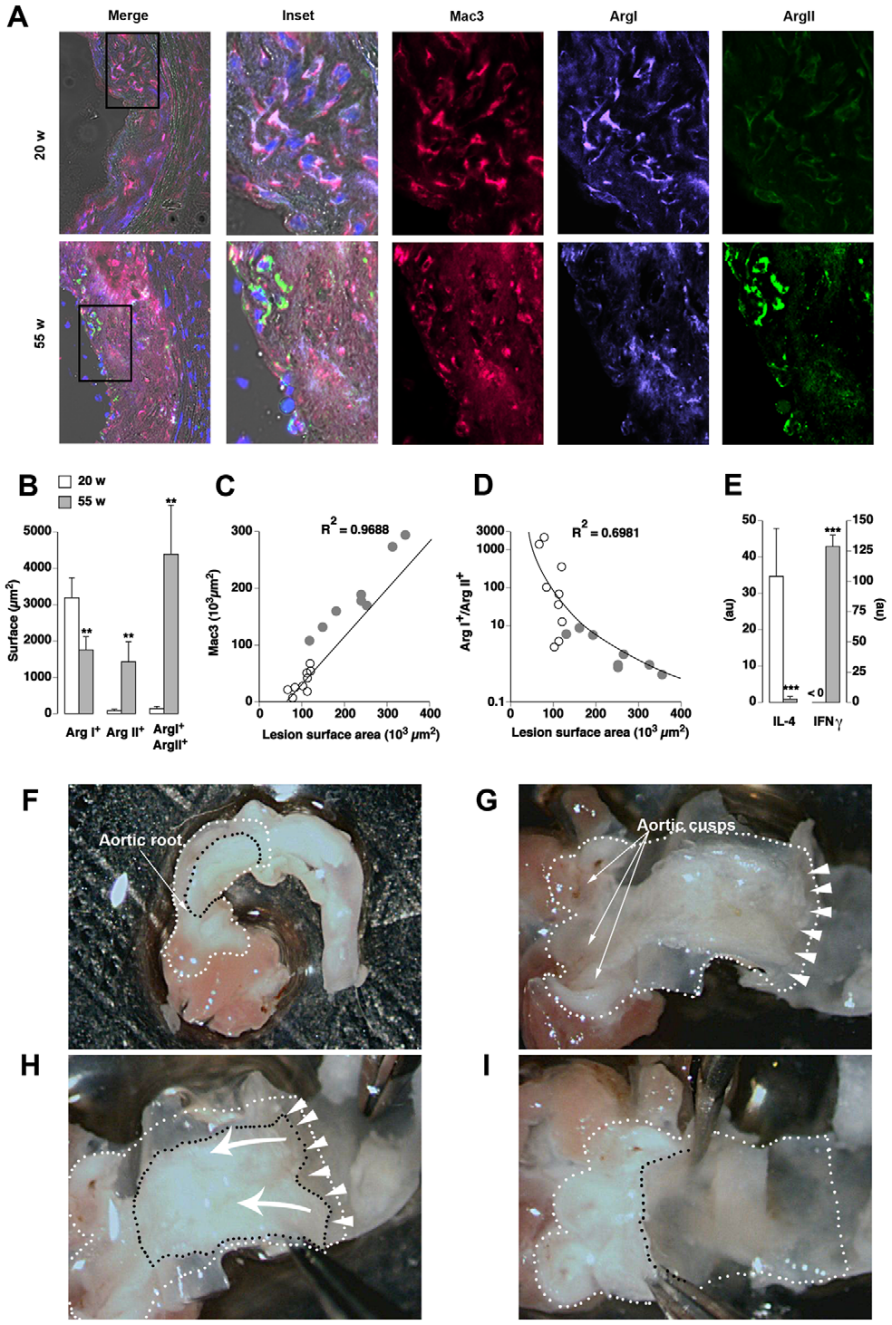
Macrophages of early atherosclerotic lesions in ApoE KO mice express Arg I while Arg II predominates in late stages. **A**: Immunofluorescence of atherosclerotic lesions from 20 and 55 week-old ApoE KO mice. MØ were identified as Mac3^+^ (red) cells. Co-expression of Mac3 and Arg I (violet) and/or Arg II (green) were identified by image overlay. Merge: overlay of bright field, DAPI, Mac3, Arg I and Arg II stainings. Inset: magnification of the zone delimited in the “Merge” frame. **B**: Arg I, Arg II and Arg I^+^ Arg II^+^ double positive surface areas within 3 random fields/plaque of 20 (white) and 55 (grey) week-old mice. **C, D**: Regression analysis between the surface area of plaques (X-axis) and total MØ area (Mac3, C) or the Arg I^+^/Arg II^+^ ratio (D). R^2^: regression correlation coefficient. **E**: Expression of IL-4 and IFNγ transcripts on microdissected atherosclerotic lesions from 20 and 55 week-old ApoE KO mice determined by real time PCR and normalized by HPRT (au: arbitrary unit). **; ***: p<0.001; p<0.0001 vs 20 w. **F–I**: Microdissection of aortic atherosclerotic lesions. Lesions can be detected (delimited by black dashes) through the vascular wall in the dissected aortic root (white dashes). The vascular wall was opened longitudinally to expose the luminal side of the vessel (G). The aortic cusps are readily identified. Arrow heads indicate the plane of fracture at the lesion/media interface, which allows the separation of the lesion from the media. The arrows in H indicate the movement performed with tweezers to detach the lesion. Appearance of the vessel after the dissection of the lesion is shown in I.

Since the two isoforms of the same enzyme appeared mutually exclusive of M1 or M2 phenotype (**Fig. 2**), immunofluorescent analysis of Arg I and Arg II expression (**Fig. 3A**) was used to identify respectively M2 and M1 type MØ (Mac3^+^ cells) in aortic root cryosections from 20 week-old (fatty streaks) and 55 week-old (advanced plaques) ApoE KO mice.

At 20 weeks of age, lesions contained virtually only Arg I^+^ MØ (**Fig. 3B**). At 55 weeks, the situation changed dramatically as a considerable number of Arg II^+^ and Arg I^+^ Arg II^+^ double positive MØ were detected (**Fig. 3B**). As expected, the surface area occupied by MØ (Mac3^+^) was directly proportional to lesion size (**Fig. 3C**). When considering the relative abundance of M2 over M1 MØ (Arg I^+^ single positive/Arg II^+^ single positive ratio) it appeared that, regardless of the disease stage, the prevalence of the M2 phenotype was associated with smaller plaque surface areas (**Fig. 3D**).

In order to explore possible changes in the cytokine microenvironment during disease progression, the transcription levels of a pro-M2 cytokine, IL-4 [9], and of a pro-M1 cytokine, IFNγ [2], were quantified by real time PCR on reverse-transcribed RNA extracted from microdissected atherosclerotic lesions (**Fig. 3F–I**) from 20 and 55 week-old ApoE KO mice.

As shown in **Fig. 3E**, IL-4 was the predominant transcript in the atherosclerotic lesions of 20 week-old ApoE KO mice but it decreased dramatically at 55 weeks of age. In contrast, IFNγ expression was undetectable in plaques of 20 week-old mice whereas it became predominant in 55 week-old mice (**Fig. 3E**).

### Effect of ApoE on the Macrophage Expression of PPARγ

**Figure 4:**
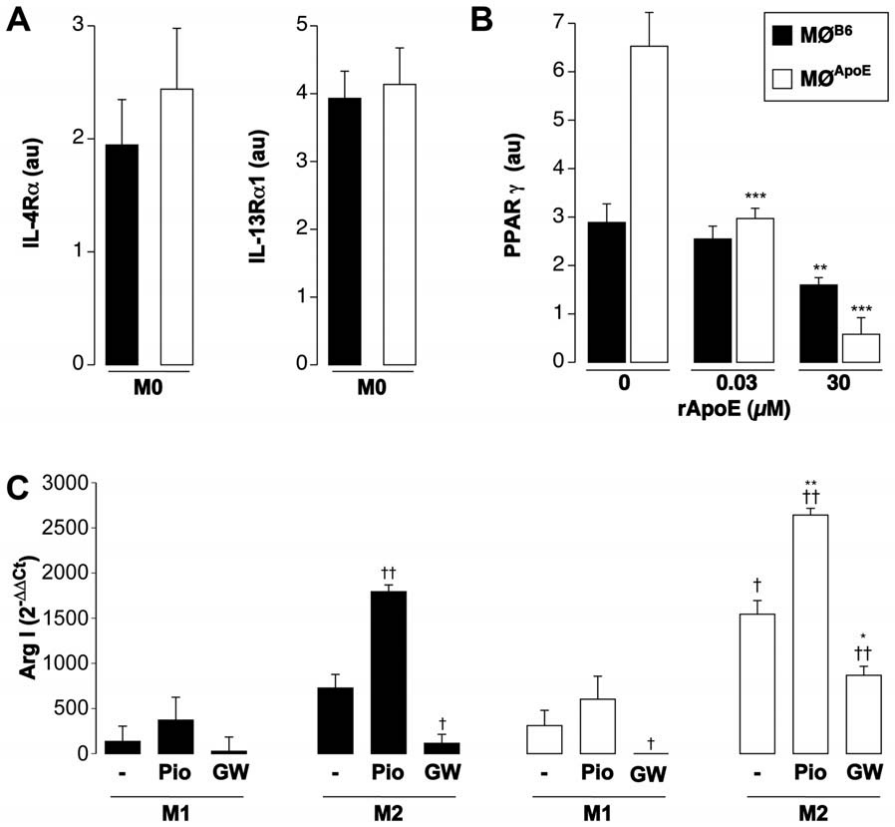
ApoE modulates the expression of PPARγ. **A**: Expression of IL-4Rα1 and of IL-13Rα1 in non-polarized MØ^B6^ and MØ^ApoE^ determined by real time PCR and normalized by HPRT (au: arbitrary unit). **B**: Expression of PPARγ in non-polarized MØ from MØ^B6^ (black) and MØ^ApoE^ (white) mice with increasing doses of recombinant ApoE (rApoE) determined by real time PCR and normalized by HPRT (au: arbitrary unit). **: p<0.001; ***: p<0.0001 vs MØ^B6^. **C**: Expression of ArgI in M1 and M2 MØ^B6^ (black) and MØ^ApoE^ (white) mice polarized with or without (−) pioglitazone (Pio) or GW9662 (GW) determined by real time PCR. The Results are expressed as 2^−ΔΔCt^ [30]. *: p<0.05; **: p<0.001 vs matched MØ^B6^ condition; †: p<0.05; ††: p<0.01 vs matched M1 condition.

The more pronounced M2 potential of MØ^ApoE^ suggested that the response to IL-4 could be heightened in the absence of ApoE. IL-4 can signal through two heterodimeric receptors composed of the IL-4Rα chain complexed with either the common γc chain or the IL-13Rα1 chain. As shown in **Fig. 4A**, the expression of IL-4Rα and of IL-13Rα1 were similar in non-polarized MØ from the two genotypes. This ruled out the possibility that the enhanced response to IL-4 was dependent upon a distinct basal receptor expression in MØ^ApoE^.

We next analyzed the expression level of transcription factors downstream to the IL-4 receptor, in particular that of peroxisome proliferator-activated receptor, PPARγ, a member of the nuclear receptor superfamily of transcription factors binding to fatty acids and derivatives of cholesterol. We found that PPARγ transcripts were significantly increased in non-polarized MØ^ApoE^ as compared to MØ^B6^ (**Fig. 4B**). The addition of exogenous ApoE reduced the level of PPARγ expression both in MØ^ApoE^ and MØ^B6^ (**Fig. 4B**). As shown in **Fig. 4C**, addition of a PPARγ synthetic agonist, pioglitazone, further increased the expression of Arg I in M2 MØ. Medium conditioned by these M2-polarized MØ in the presence of pioglitazone increased the proliferation of VSMCs (**Fig. 1B**). Conversely, inhibition of PPARγ by GW9662 significantly reduced the expression of Arg I, regardless of the polarization condition, but to a lesser extent in M2 MØ from ApoE KO mice (**Fig. 4C**). PPARγ inhibition also reduced the pro-mitotic properties of M2 MØ-conditioned medium on VSMCs (**Fig. 1B**). Of note, a selective arginase inhibitor, Nor-NOHA, also significantly reduced the pro-mitotic activity of MØ- conditioned medium (**Fig. 1B**) indicating that arginase activity contributes to the pro-mitotic activity of MØ.

### ApoE-Deficient Macrophages Retain Their Plasticity

**Figure 5:**
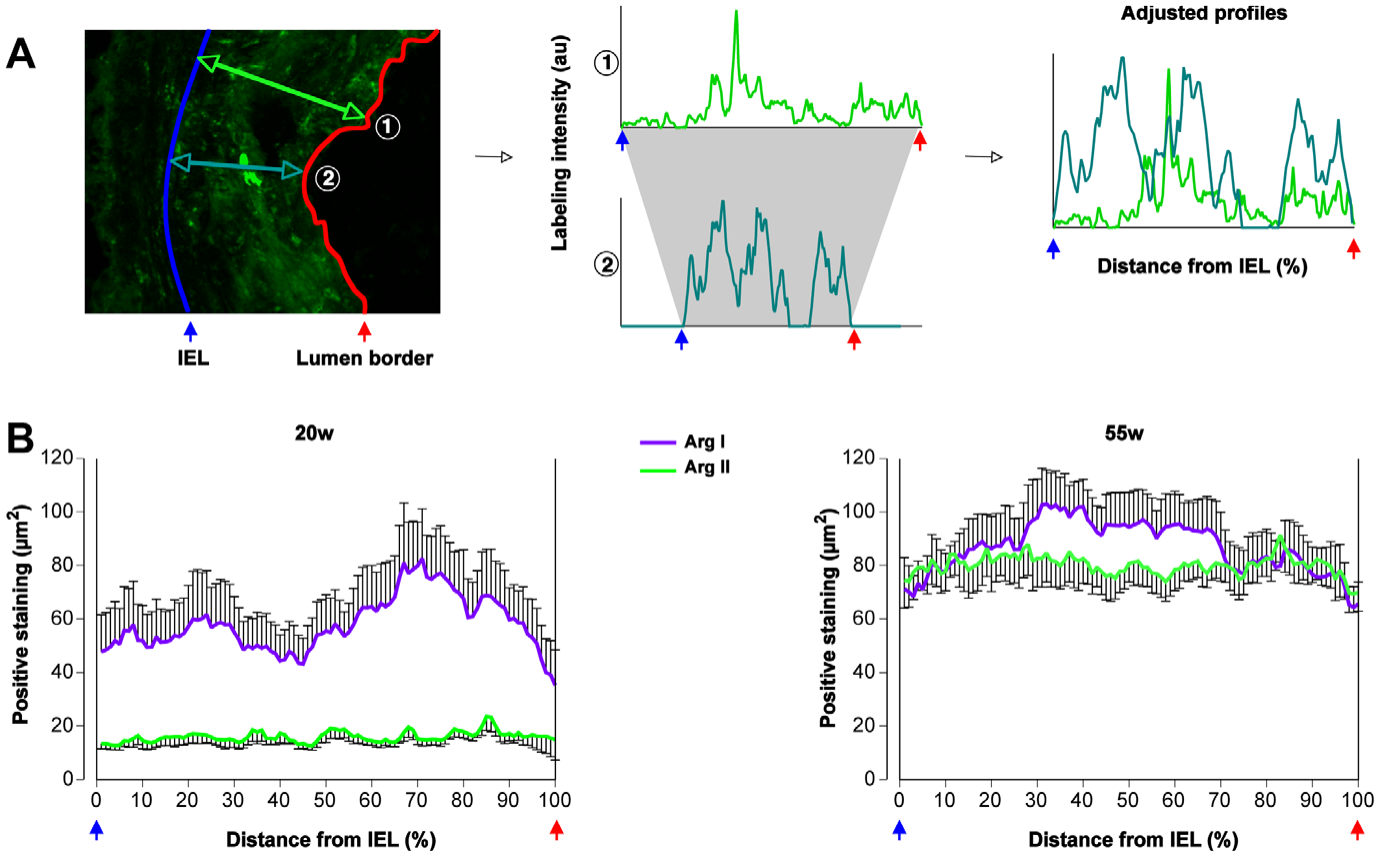
Distribution of Arg I^+^ and Arg II^+^ macrophages in atherosclerotic lesions. **A**: A new morphometric method was set up to analyze the distribution of Arg I^+^ and Arg II^+^ MØ based on profiles (n = 10 per lesion, 3 lesions per mouse) outlined accross the lesions between the internal elastic lamina (IEL) and the lumen border. To compare the profiles regardless of the lesion thickness, they were adjusted and represented according to the percentage of the IEL-lumen border distance. Two profiles 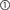 and 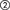 are plotted as representative examples. **B**: Adjusted profiles showing the Arg I and Arg II labeling distributions in the lesion in early (20w) and advanced (55w) aortic cusps of ApoE KO mice.

In spite of the intrinsic M2 bias of MØ from ApoE KO mice, a consistent number of Arg II^+^ (M1) MØ were detected in advanced atherosclerotic lesions. This could be due to the recruitment of MØ of different phenotypes during the course of the disease. We therefore evaluated the location of Arg I and of Arg II labeling within the plaque using a new quantification method (**Fig. 5A**), assuming that newly recruited MØ would reside in the luminal side of the plaque. While Arg I^+^ (M2) MØ accumulated in the luminal side of the plaques in young mice, at 55 weeks of age, both Arg I and Arg II labeling was evenly distributed across the plaque (**Fig. 5B**) arguing against a sequential recruitment of MØ with distinct phenotypes. Alternatively, the enrichment of advanced atherosclerotic plaques in M1 MØ could be due to a phenotypic conversion of plaque-infiltrated MØ. We therefore tested whether, in the absence of ApoE, *in vitro* polarized MØ retain their plasticity. Bone marrow-derived MØ were first polarized into M1 or M2 and subsequently subjected to a second and opposite stimulation.

**Figure 6:**
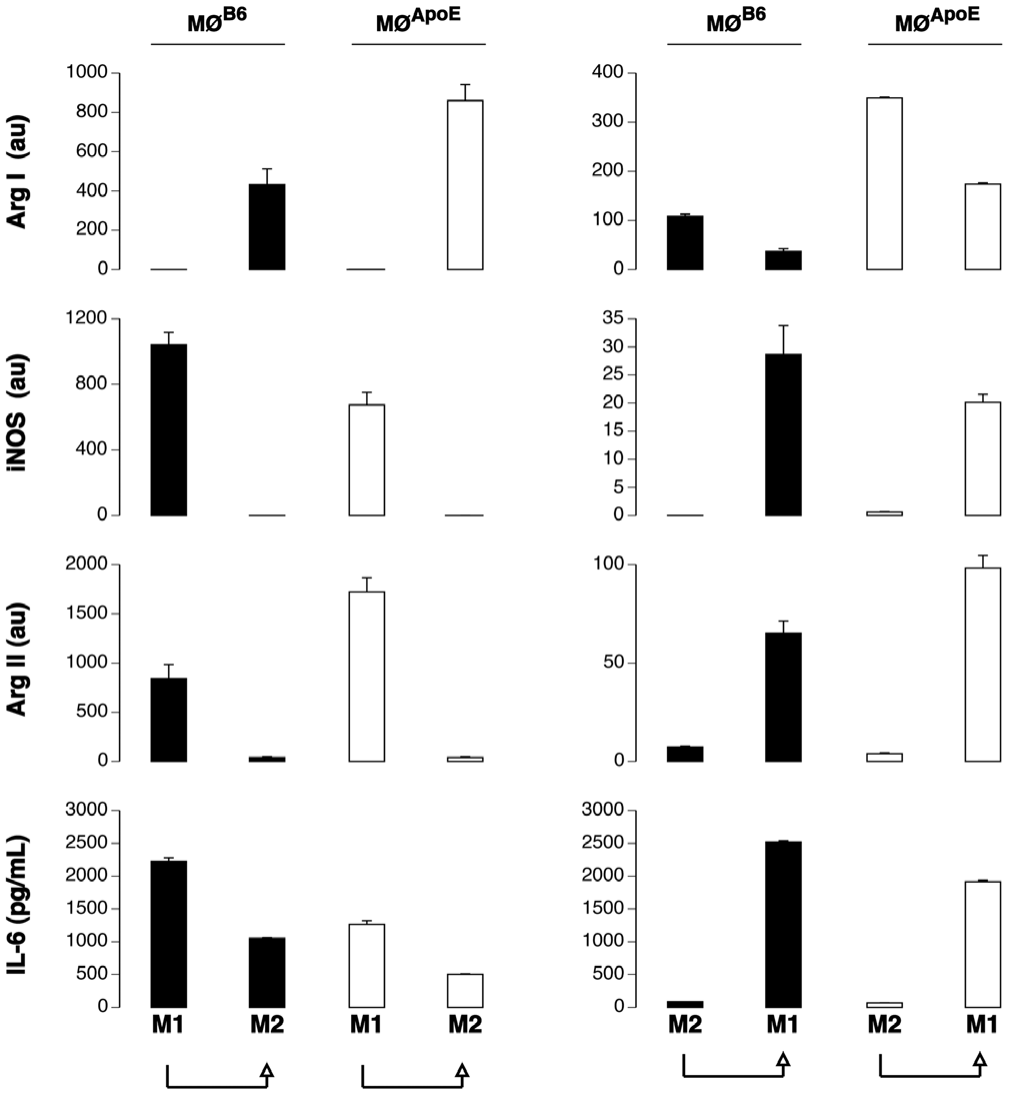
Preserved plasticity of fully polarized macrophages. MØ^ApoE^ (white) and MØ^B6^ (black) were primed and subjected to a first M1 or M2 polarization. After ten hours, the culture conditions were switched during 10 additional hours in order to induce the opposite phenotype. Expression of iNOS, Arg I and ArgII was evaluated by real time PCR and normalized by HPRT (au: arbitrary unit). Results are representative of three distinct experiments.

As in MØ^B6^ mice, fully polarized MØ from ApoE KO mice completely reverted from their the initial phenotype to the opposite one after the second stimulation. After 10 h, M1 MØ cultured with IL-4 turned on the expression of Arg I while the expression of iNOS, Arg II and production of IL-6 were shut down (**Fig. 6** **, left**). On the other hand, M2 MØ stimulated with LPS/IFNγ increased their expression of iNOS, Arg II, and IL-6 while Arg I expression was considerably blunted (**Fig. 6** **, right**).

Therefore fully polarized MØ retained their plasticity regardless of the absence of ApoE.

## Discussion

It is well established that MØ play a key role in atherosclerosis [1]. The present study corroborates this paradigm since we found a direct correlation between the number of plaque-infiltrated MØ and the size of the lesion. Such a positive correlation can be interpreted as evidence for a pro-inflammatory and pathogenic role of MØ in atherosclerosis. However, MØ are very versatile cells with a high degree of plasticity in response to a range of environmental signals. Classically (M1) and alternatively (M2) activated MØ represent the two extremes of a continuum of MØ activation states, some of which can exert a beneficial effect [3]. In the present study, we found that Arg I and Arg II were distinctly upregulated in bone marrow-derived M1 and M2 MØ and used them as surrogate markers of M1 and M2 MØ to evaluate whether a particular phenotype of MØ is associated with the progression of atherosclerotic disease.

### Plaque-Infiltrated Macrophages Are Initially of the Reparative Phenotype

Analysis of plaque-infiltrated MØ in young ApoE KO mice revealed a uniform Arg I^+^ (M2) MØ phenotype. Concomitantly, lesions consistently contained large amounts of IL-4. The source of IL-4 at these sites could be either neutrophils [10] or NKT cells [11], as suggested by recent studies showing the important contribution of these cells to atherogenesis [12], [13], [14]. The unexpected association of M2 reparative MØ with the initial stages of atherogenesis does not necessarily imply that this MØ phenotype contributes to the disease progression. Alternatively, we propose that the M2 MØ exert a reparative action in atherosclerosis since M2-conditioned medium promoted the proliferation of vascular cells *in vitro*.

### PPARγ Favors the Reparative Macrophage Phenotype

*In vitro* polarization studies showed that the lack of ApoE favors the reparative M2 phenotype in response to IL-4. The more pronounced M2 potential of ApoE KO MØ was not due to increased availability of the receptor to IL-4 but rather to an increased expression of peroxisome proliferator-activated receptor (PPAR)γ, a major transcription factor downstream to the IL-4 receptor [15] and an emerging key regulator of MØ differentiation, linking lipid metabolism and inflammation [16]. Addition of a PPARγ agonist (pioglitazone) further increased the expression of Arg I of ApoE KO MØ in response to IL-4. Conversely, PPARγ inhibition by GW9662 reduced the expression of Arg I. In agreement with previous studies performed on human MØ [17], we propose that PPARγ sustains the M2 activation pathway of MØ in mice.

Interestingly, the addition of exogenous ApoE down-regulated, in a dose-dependent manner, the levels of PPARγ expression in ApoE KO MØ and restored a feedback control exerted by ApoE on this transcription factor. Indeed, this effect was also found in MØ from wildtype mice, suggesting that ApoE exerts a physiological control over PPARγ transcription.

### The Pro-Mitotic Activity of M2 Macrophages Is Related to Their Expression of Arginase

We found that the pro-mitotic effect of M2 MØ-conditioned medium on VSMCs was significantly reduced when MØ polarization was performed in the presence of Nor-NOHA, an arginase inhibitor, indicating that arginase activity contributes to the pro-mitotic effect of M2 MØ. The experiments performed with medium conditioned by MØ polarized in presence of pioglitazone or of GW9662 corroborate this statement. Indeed, medium conditioned by MØ polarized with pioglitazone, expressing high levels of Arg I, induced a strong proliferation of VSMCs. Instead, the proliferation of VSMCs was reduced when these cells were cultured with medium conditioned by MØ polarized with GW9662 expressing low levels of Arg I. The reason why M1 MØ -conditioned medium failed to promote VSMC proliferation is puzzling since M1 MØ express Arg II, an enzyme that catalyzes the same reaction as Arg I. Given that iNOS and arginases use L-Arg as a common substrate and given their respective Km (∼5 µmol/L vs 10 mmol/L) and Vmax values (10^3^–10^4^ times higher for arginases), these enzymes are expected to compete for L-Arg. Hence, iNOS may down-regulate proline and polyamine production in M1 MØ by competing with Arg II for L-Arg. The change of the arginase isoform according to polarization could allow each MØ type to distinctly address arginases to the cytosolic (Arg I) or mitochrondrial (Arg II) subcellular compartments where they may exert distinct biological effects as reported in endothelial cells [18].

### Macrophage Phenotypic Switch Correlates with Lesion Progression

With disease progression, Arg II^+^ (M1) MØ prevailed over the Arg I^+^ (M2) MØ. Regression analysis showed that the M2/M1 ratio of plaque-infiltrated MØ is inversely proportional to the progression of lesion size in apoE KO mice, indicating that plaque growth is linked to the enrichment in pro-inflammatory M1 MØ.

The enrichment of the advanced atherosclerotic plaque in Arg II^+^ (M1) MØ could be due to a phenotypic conversion of plaque-infiltrated, polarized MØ. This hypothesis is supported by the fact that Arg I can be co-detected with Arg II (double positive staining) in MØ present in advanced plaques. This finding is reminiscent of what we found *in vitro*: M2 cells repolarized to the M1 phenotype maintain a certain level of Arg I expression, which is absent in directly polarized M1 MØ. MØ plasticity has been extensively described in wildtype cells [19], [20], [21]. While IFNγ promotes the M1 phenotype in MØ, IL-4 induces the M2 phenotype [2], [22]. Importantly, the plaque cytokine microenvironment changed over time, since we documented a local increase in the expression of IFNγ at the expense of IL-4 within advanced atherosclerotic plaques. Alternatively, the change in the phenotype of plaque infiltrating MØ in advanced lesions could result from newly recruited M1 MØ. If this were the case, one would expect M1 cells to be present mainly in the luminal side. We found that both Arg I and Arg II labeling was evenly distributed across the advanced lesions. These data thus do not support a sequential recruitment of M2 and of M1 MØ over time. We however cannot definitively rule out the possibility that a change in the local cytokine milieu differentially polarizes new incoming monocytes. Thus, we propose that the initial MØ phenotype is of the M2 type and the changes in the cytokine milieu of the advanced plaque favor the emergence of pro-inflammatory M1 MØ, the prevalence of which is directly correlated with plaque growth.

In the present study, we used Arg I and Arg II as surrogate markers of M1 and M2 MØ since these two isoforms of arginase were distinctly upregulated in bone marrow-derived M1 and M2 MØ polarized *in vitro*. However, it has been shown that IFNγ and LPS differently modulate Arg II expression in M1 MØ [8] and that Arg I can be expressed by MØ that have been activated independently of M2-polarizing conditions (IL-4/IL-13) [23], [24]. Therefore, we cannot formally exclude that Arg I^+^ and Arg II^+^ MØ observed *in vivo* may also reflect MØ activation states that differ from the ones obtained by the polarization conditions that we used *in vitro*.

### Macrophage Plasticity in Atherosclerosis: Pathophysiologic and Therapeutic Considerations

The phenotypic change of plaque MØ might depend upon the type (innate or adaptative) of the immune effectors infiltrating the developing lesion. It is of interest that a recent study by Loke et al. showed that the initial M2 MØ phenotype at the site of inflammation is independent of the cells of the adaptive immune system whereas the maintenance of the M2 MØ phenotype requires IL-4-producing T cells, in the absence of which M2 MØ switch to the M1 phenotype [25]. Transposing this mechanism to atherosclerosis suggests that the Th1 phenotype might trigger the M2->M1 phenotypic switch in advanced atherosclerotic plaques, when the effectors of the innate immunity hand over the relay to the effectors of the adaptive immune system [26].

In a therapeutic perspective, controlling the local cytokine milieu within atherosclerotic plaques *in vivo* will be difficult to achieve. On the other hand, a recent histopathologic study on human carotid plaques [17], has suggested that PPARγ activation may skew MØ polarization toward the M2 phenotype. The present experimental study demonstrates that PPARγ agonists, which are already being employed in clinical practice and have shown an atheroprotective effect [27], do indeed potentiate the response to M2 polarizing agents in mouse MØ. We therefore propose that the use of PPARγ agonists could contribute to the stabilization of atherosclerotic lesions by favoring the differentiation and/or conversion of plaque-infiltrated MØ into the reparative M2 phenotype.

## Methods

### Analysis of Atherosclerotic Lesions

Immunohistochemistry and lesion morphology analysis were performed as previously described [28] on serial aortic cryosections from 20 (n = 10) or 55 (n = 10) week-old ApoE KO mice (obtained from the Jackson Laboratory and bred in our facility) maintained on a regular chow diet. Mice were handled in accordance with European Union directives (86/609/EEC) on the care and use of laboratory animals. The investigation was approved by the Animal Ethics Committee of the Institut National de la Santé et de la Recherche Médicale. The review and approval of the study was also obtained by the Local Animal Ethics Committee (No. B 7518 03).

### Immunohistology

Cryosections were fixed in acetone, delipidized with alcohol and toluene, and re-hydrated. Endogenous peroxidase and avidin/biotin were blocked using commercial Kits (DAKO). When using mouse anti-mouse antibodies, slides were pre-incubated with a F(ab)'2 fragment-goat anti-mouse IgG (Jackson immunoResearch) and the M.O.M. immunodetection Kit (Vector Laboratories). Arg I^+^ and Arg II^+^ MØ were identified with a purified rat anti-mouse Mac3 monoclonal antibody (BD Biosciences), an immunopurified rabbit anti-mouse Arg II polyclonal antibody [29], and a purified mouse anti-mouse ArgI monoclonal antibody (BD). The staining was revealed by using fluorescent secondary antibodies (Molecular Probes): Alexa Fluor 647 goat anti-rat IgG (H+L) for Mac3, Alexa Fluor 546 anti-mouse for Arg I and Alexa Fluor 488 goat anti-rabbit for ArgII. Immunostained slides were cover-mounted with Prolong Gold antifade reagent (Invitrogen). The fluorescence was detected with a Zeiss Axiovert 200 M microscope equipped with the AxioCam MRm vers.3 camera, the ApoTome® system and the AxioVision® image capture software. Images were acquired using the EC “Plan-Neofluar” 40x/0,75 (df = 0,71 mm) objective of the microscope.

In order to evaluate the spatial distribution of Arg I and Arg II labeling within the plaque, we have set up a morphometric method based on intensity profiles of each immunostain and a normalization of the distance between the internal elastic lamina (IEL) and the luminal border of the plaque. To this purpose, we have quantified by computer-assisted image analysis the profiles over 10 segments (mean convergence ≥7) crossing the plaques of each aortic cusp of each mouse (30 profiles/immunostain/mouse; **Fig. 5A**).

### Bone Marrow-Derived Macrophage Isolation and Differentiation

Primary cultures of bone marrow-derived MØ were obtained from femurs of 6–10 week-old C56BL/6 (B6, Janvier) or ApoE KO mice as described [20] and cultured in complete medium (MEM supplemented with 10% FCS, 800 pg/ml Macrophage-Colony Stimulating Factor-1 [20% L-929-conditioned medium]). Non-adherent cells were collected after 24 h and were differentiated for 7 days yielding 98% Mac3^+^, 98% F4/80^+^ and 99% CD11b^+^ cells (data not shown).

### Polarization of Bone Marrow-Derived Macrophages

Differentiated bone marrow-derived MØ were polarized with either 100 ng/ml LPS+100 U/ml IFNγ (M1) or 5 ng/ml IL-4 (M2) [2]. The effect of an overnight priming with 100 U/ml IFNγ prior to polarization [7] was also tested. Non-polarized MØ were cultured in complete medium alone.

To test the effect of PPARγ, a pharmacological agonist (pioglitazone, 1 µM) or an antagonist (GW9662, 1 µM) were added 2 hours before the polarization, which was performed as described above.

Human recombinant ApoE (rApoE) was desalted to eliminate the toxic ammonium bicarbonate and was used at 0.03 and 30 µM.

For MØ plasticity studies, cells were cultured in the pro-M1 conditions described above for 10 hours, washed and then submitted for an additional 10 hours to the pro-M2 environment (M1 ->M2). The experiment was mirrored to study the M2 ->M1 switch.

At the end of the stimulation, culture supernatants were collected and stored at −80°C prior to IL-6 analysis (ELISA, BD Biosciences). MØ were washed and lysed in Trizol for RNA extraction and analysis of gene expression.

### Gene Expression Studies

Total RNA from cells or atherosclerotic lesions microdissected from the ascending aorta just above the aortic cusps (**Fig. 3F–I**) was isolated and reverse-transcribed using Superscript II reverse transcriptase. Real time PCR was performed on cDNA with the primer pairs listed in **Table 1** on a CFX 100 (Biorad) or a SDS7700 (Applied Biosystems) cycler (2 µl cDNA, 250 nM primers, 11 µl Syber-Green master mix from Qiagen; 1 cycle: 50°C, 2 min, 1 cycle: 95°C 15 min, and 60 cycles: 95°C 40 s, 60°C 1 min). Dissociation curve analysis was performed at the end of 60 cycles to verify the identity of the PCR product. No signals were detected in no-template controls. When results concerning the three M0, M1 and M2 conditions were required, 4 serial ¼ dilutions of a cDNA control sample were used to establish a standard curve in each reaction. Expression of genes of interest was normalized to the level of expression of the constitutively expressed hypoxanthine-guanine phosphoribosyltransferase (HPRT) gene and are expressed as arbitrary units (au). When M1 and M2 conditions were expressed as compared the M0 reference, the 2^−ΔΔCt^ formula as described by Pfaffl MW [30] was applied, where threshold cycle “Ct” values correspond to the cycle at which PCR enters the exponential phase.

**Table 1:** List of primers.

Gene	Forward primer	Reverse primer
miNOS	TGCATGGACCAGTATAAGGCAAGC	GCTTCTGGTCGATGTCATGAGCAA
mArg I	CAGAAGAATGGAAGAGTCAG	CAGATATGCAGGGAGTCACC
mArg II	TGATTGGCAAAAGGCAGAGG	CTAGGAGTAGGAAGGTGGTC
mHPRT	CCTGCTGGATTACATTAAAGCACTG	GTCAAGGGCATATCCAACAACAAAC
mIL-4Rα	AGTGAGTGGAGTCCTAGCATC	GCTGAAGTAACAGAACAGGC
mPPARγ	CGAGAAGGAGAAGCTGTTGG	GAAACTGGCACCCTTGAAAA
mIL-4	GCTTTGCAGCTCTTCCTCAT	CTTTTGCCAGTTCCTCCA
mIFNγ	ACCATTCCAGTCTTTGTCGC	CAGGATCAGGAATTGGAGGA
mIL-13Rα1	ACCATTCCAGTCTTTGTCGC	CAGGATCAGGAATTGGAGGA
mYm1/2	CAGGGTAATGAGTGGGTTGG	CACGGCACCTCCTAAATTGT

### Arterial VSMC Proliferation

Primary vascular smooth muscle cells were obtained from explant cultures of aortic tissue from C57BL/6 mice as described [31] without the enzymatic digestion step. The full length of the thoracic aorta was aseptically dissected and the adventitia was removed. The aorta was cut into small segments (∼1 mm^3^), which were incubated for 1 h at 37°C in 5% CO_2_ in a drop of 10% FCS DMEM and then cultured in standard conditions for 2 weeks.

VSMCs were trypsinized, seeded at an initial density of 5,000 cells/well in 96-well plates and grown to sub-confluence (2 days). Cells were then left quiescent for 48 h in 1% FCS DMEM. Culture medium conditioned by M0, M1, or M2 MØ, harvested 10 h after polarization, were added to the VSMCs for 48 h. In certain wells, the medium was conditioned by MØ polarized in the presence of 50 µM or 100 µM Nor-NOHA, a specific arginase inhibitor or in the presence of 1 µM pioglitazone or 1 µM GW9662. Control wells included VSMCs cultured in the presence of 100 ng/ml LPS+100 U/ml IFNγ,5 ng/ml IL-4, 50 or 100 µM Nor-NOHA, 1 µM pioglitazone, or 1 µM GW9662 in serum free medium (BSA 4%) to test whether the molecules used during the polarization of MØ still remaining in the MØ-conditioned medium had a direct effect on cell proliferation. At the end of the assay, the number of viable cells in each condition was evaluated by the reduction of the dye 3-[4,5-dimethylthiazol-2-yl]-2,5 diphenyltetrazolium (MTT tetrazolium, 0.5 mg/ml final concentration in wells) [32] and by using a standard curve established with known numbers of cells (8 wells ranging from 1000 up to 80 000 VSMCs). MTT was incubated for 3 hours at 37°C, the medium removed, and the formazan product dissolved in 100 ml DMSO. Product formation was assessed spectrophotometrically by measuring the OD at 590 nm.

### Statistical Analysis

Results are expressed as means ± SEM. Differences between groups were evaluated by one-way ANOVA. Differences were considered significant when p<0.05. Correlations between continuous variables were calculated by regression. Statistical analysis was performed using Statview 5.0 Software (SAS Institute Inc., USA).
